# Anti-glaucoma potential of *Heliotropium indicum* Linn in experimentally-induced glaucoma

**DOI:** 10.1186/s40662-015-0027-1

**Published:** 2015-10-01

**Authors:** Samuel Kyei, George Asumeng Koffuor, Paul Ramkissoon, Osei Owusu-Afriyie

**Affiliations:** Discipline of Optometry, School of Health Sciences, College of Health Sciences, University of KwaZulu- Natal, Durban, South Africa; Department of Optometry, School of Physical Sciences, University of Cape-Coast, Cape-Coast, Ghana; Department of Pharmacology, Faculty of Pharmacy and Pharmaceutical Sciences, Kwame Nkrumah University of Science and Technology, Kumasi, Ghana; Department of Pathology, Komfo Anokye Teaching Hospital, Kumasi, Ghana

**Keywords:** Glutathione assay, Steroid-induced glaucoma, Acetazolamide, New Zealand white rabbit, Anti-glaucoma drug, Glutamate

## Abstract

**Background:**

*Heliotropium indicum* is used as a traditional remedy for hypertension in Ghana. The aim of the study was to evaluate the anti-glaucoma potential of an aqueous whole plant extract of *H. indicum* to manage experimentally-induced glaucoma.

**Methods:**

The percentage change in intraocular pressure (IOP), after inducing acute glaucoma (15 mLkg^−1^ of 5 % dextrose, i.v.), in New Zealand White rabbits pretreated with *Heliotropium indicum* aqueous extract (HIE) (30–300 mgkg^−1^), acetazolamide (5 mgkg^−1^), and normal saline (10 mLkg^−1^) *per os* were measured. IOPs were also monitored in chronic glaucoma in rabbits (induced by 1 % prednisolone acetate drops, 12 hourly for 21 days) after treatments with the same doses of HIE, acetazolamide, and normal saline for 2 weeks. The anti-oxidant property of the extract was assessed by assaying for glutathione levels in the aqueous humour. Glutamate concentration in the vitreous humour was also determined using ELISA technique. Histopathological assessment of the ciliary bodies was made.

**Results:**

The extract significantly reduced intraocular pressure (*p* ≤ 0.05–0.001) in acute and chronic glaucoma, preserved glutathione levels and glutamate concentration (*p* ≤ 0.01–0.001). Histological assessment of the ciliary body showed a decrease in inflammatory infiltration in the extract and acetazolamide-treated group compared with the normal saline-treated group.

**Conclusion:**

The aqueous whole plant extract of *Heliotropium indicum* has ocular hypotensive, anti-oxidant and possible neuro-protective effects, which therefore underscore its plausible utility as an anti-glaucoma drug with further investigation.

## Background

Glaucoma, referred to as the silent thief of sight, is recorded as the second most important cause of blindness and the leading cause of irreversible blindness globally [1, 2].

It is said to be a heterogeneous group of diseases resulting from multiple causative factors including increase in intraocular pressure (IOP) and vascular dysregulation. These factors largely contribute to the initial injury in this disorder by hindering axoplasmic flow within the retinal ganglion cell (RGC) axons at the lamina cribrosa, impairing the optic nerve microcirculation at the level of lamina, and changing the laminar glial and connective tissue [3]. Factors leading to further damage include excitotoxicity caused by glutamate or glycine that is freed from injured neurons and oxidative damage [4]. Despite the provision of appropriate treatment, blindness still occurs in nearly 10 % of sufferers [5]. The most common form of the glaucomas, primary open angle glaucoma (POAG), presents with no warning symptoms, especially at its early stages [6].

Ghana is one of the worse affected countries in the world as it is ranked second after St. Lucia in terms of glaucoma prevalence [7]. It is also reported to have early age of onset (30 years) compared to the global trend of 40 years, with risk factors such as age and ethnicity [8–10]. The most aggressive form of glaucoma has been reported among people of African descent and they are three times more likely to suffer from glaucoma compared to Caucasians [11]. Cost-of-illness studies have shown the importance of this disease, with the United Kingdom spending more than £300 million in 2002 on glaucoma prevention and treatment [12]. In the United States, it is the reason for over 10 million visits to physicians annually, with a yearly estimated cost of over $1.5 billion to its government [5]. Elsewhere in Africa where there is reliable data, it is evident that the middle-class spent more than half their monthly income, while low-income earners spent virtually all their monthly take-home salary to treat glaucoma [13]. This makes it an expensive disease so far as its management is concerned.

Management options for glaucoma include the use of medicines, as well as lasers and incisional surgery, with medical therapy being the most common [14]. None of these management procedures are free of complications, with some leading to loss of vision instead of its preservation [15, 16]. The development of new treatment options with minimal side effects is therefore important, specifically those that target the modifiable pathogenic factor of ocular hypertension in addition to others.

It is within this context that the current study investigated the anti-glaucoma potential of an aqueous whole plant extract of *Heliotropium indicum* L. (Boraginaceae) also known as cock’s comb to manage experimentally-induced glaucoma as an initial step in bioprospecting for treatment options for the disease. In Ghana and elsewhere in Africa, *H. indicum* is widely used as a traditional remedy for several diseases such as abdominal pain, convulsion, cataract, conjunctivitis, cold and high blood pressure among others [17, 18]. The plant is prepared and applied in various forms such as decoction, powder, cold infusion, poultice, concoction or squeezing its juice onto the affected area depending on the ailment. In some localities in Ghana, it is used in preparation of soup for postpartum women to treat inflammatory reactions. For the purposes of pressure-lowering effect, preparations of *H. indicum* are used orally as a decoction, concoction or as a dietary ingredient in locally prepared soups.

## Methods

### Plant collection

*Heliotropium indicum* was collected in November, 2012, from the University of Cape Coast botanical gardens (5.1036° N, 1.2825° W), located in the Central Region of Ghana. It was identified and authenticated by a botanist at the School of Biological Sciences, College of Agricultural and Natural Sciences, University of Cape Coast, Cape Coast, Ghana. A voucher specimen, numbered 4873, has been deposited at the herbarium.

### Preparation of the *H. indicum* aqueous extract (HIE)

Whole plants of *H. indicum* were washed thoroughly with tap water and shade-dried. The dried plants were milled into coarse powder (1.5 kg) by a hammer mill (Schutte Buffalo, New York, NY), then mixed with 1 liter of water. The mixture was soxhlet extracted at 80 °C, for 24 h, and the aqueous extract was freeze-dried (Hull freeze-dryer/lyophilizer 140 SQ, Warminster, PA). The powder obtained (yield 12.2 %), was labelled HIE, and stored at a temperature of 4 °C. This (HIE) was reconstituted in normal saline to the desired concentration for dosing in this study.

### Drugs and chemicals used

Prednisolone acetate ophthalmic suspension (1 %) (Alcon Laboratories, Inc. Texas, USA) was used to induce ocular hypertension. Proparacaine hydrochloride ophthalmic solution (Ashford Laboratories Ltd, China Macau) was used as a local anaesthetic in the eyes during IOP measurements. Acetazolamide (Ernest Chemists Ltd, Tema, Ghana) was used as the reference anti-glaucoma drug.

### Experimental animals and husbandry

Twenty five New Zealand White rabbits, weighing 1.0 ± 0.2 kg, were housed singly in aluminium cages (34 cm × 47 cm × 18 cm) with soft wood shavings as bedding, under ambient conditions (temperature 28 ± 2 °C, relative humidity 60–70 %, and a normal light–dark cycle) in the Animal House of the School of Biological Sciences, University of Cape Coast, Ghana. They were fed on a normal commercial pellet diet (Agricare Ltd, Kumasi, Ghana) and had access to water *ad libitum*.

### Ethical and biosafety considerations

The study protocol was approved by the Institutional Review Board on Animal Experimentation, Faculty of Pharmacy and Pharmaceutical Sciences, Kwame Nkrumah University of Science and Technology, Kumasi, Ghana (Ethical clearance number: FPPS/PCOL/0030/2013). All activities performed during the study conformed to acceptable principles on the use and care of laboratory animal (EU directive of 1986: 86/609/EEC), and the association for research in vision and ophthalmology statement for use of animals in ophthalmic and vision research. Biosafety guidelines for protection of personnel in the laboratory were also observed.

### Preliminary phytochemical screening

Screening was performed on HIE to ascertain the presence of phytochemicals using standard procedures described by Harborne [19] and Kujur *et al.* [20].

### Assessing hypotensive effect of HIE in an acute glaucoma model

The basal IOP in each eye of each rabbit was measured using an improved Schiotz indentation tonometer (J. Sklar Manufacturing Company, Long Island City, N.Y), which was calibrated by an open manometric calibration procedure as described elsewhere [21]. Care was taken to prevent the nictitating membrane from coming under the base of the tonometer. Tension was recorded each time by two weights (5.5 g and 10 g), and the mean of the two recordings was calculated. The animals were then put into five groups (*n* = 5) labelled A-E. Groups A, B, and C received 30, 100 and 300 mgkg^−1^ HIE respectively, while Groups D and E received 5 mgkg^−1^ acetazolamide and 10 mLkg^−1^ normal saline respectively. All administration was by mouth using an oral gavage. This was to mimic its ethno-pharmacological use. Each animal received not more than 1 mL of HIE. After 30 min, 15 mLkg^−1^ of 5 % dextrose solution was administered intravenously, through the marginal ear vein. IOP measurements were made every 20 min for 120 min in each eye. The percentage change in IOPs was then determined by the following formula:

$$ \%\ \mathbf{Change}\ \mathbf{in}\ \mathbf{I}\mathbf{O}\mathbf{P} = \left(\mathbf{IO}{\mathbf{P}}_{\mathbf{t}}\hbox{--}\ \mathbf{IO}{\mathbf{P}}_{\mathbf{0}}/\ \mathbf{IO}{\mathbf{P}}_{\mathbf{0}}\right) \times \mathbf{100} $$

Where IOP_t_ is the ocular tension (at different times) after dextrose or steroid (prednisolone) administration and IOP_o_ is the ocular tension before dextrose or steroid (prednisolone administration (i.e. time zero).

### Assessing the hypotensive effect of HIE in a chronic glaucoma model

- *Induction of ocular hypertension in rabbits*
  After baseline measurements of IOPs, ocular hypertension was induced in rabbits by instilling 1 % prednisolone acetate in each eye, twice daily (12 hourly) for 21 days, while measuring the IOP weekly (between 8.30 and 9.00 AM). Animals with at least a 50 % increase in IOP and characterized with one or more of the following clinical signs: bulging eyeball (buphthalmic eyes), fixed dilated pupils, sluggish pupillary reaction, and limbal injection [22], were selected for this study.
- *Assessment of ocular hypotensive effect of HIE*
  Rabbits with ocular hypertension were divided into five groups labelled I-V. Each group was treated orally, twice daily (12 hourly), with 30, 100, 300 mgkg^-1^ HIE, 5 mgkg^−1^ Acetazolamide (positive control), or 10 ml/kg normal saline (negative control), for 2 weeks with intraocular pressure measurements being made in each eye every other day for the same period.

### Determination of glutathione in aqueous humour

Total glutathione in the aqueous humour of the experimental animals was determined using a commercial kit (Cayman Chemicals, Ann Arbor, MI, USA). The animals were euthanized and the anterior chamber punctured with a 30-gauge needle. The aqueous humour was collected from both eyes and stored in sterile eppendorf tubes. The aqueous humour was then deproteinated using metaphosphoric acid and 4 M triethanolamine according to the manufacturer’s instruction. A 50 μL volume of the deproteinated aqueous humour and the standards (constituted per the manufacturer’s directive) were pipetted into a 96 well plate, incubated in the dark on an orbital shaker, and read at 405 nm using a URIT-660 microplate reader (URIT Medical Electronic Co., Ltd, Guangxi, China). Each determination was performed in duplicates.

### Evaluation of glutamate in vitreous humour

Glutamate concentration in the vitreous humour of experimental animals was determined using the glutamate assay kit. The vitreous humour in each eye was collected in separate sterile eppendorf tubes after accessing it through a scleral puncture at the lateral canthus. The vitreous bodies were sonicated in 0.2 M perchloric acid containing 0.1 % Na_2_S_2_0_5_ and 0.1 % EDTA. Homogenates were centrifuged at l5,000 g for 5 min at 4 °C and the supernatant were used for the glutamate concentration assay. The samples and standards were prepared according to manufacturer’s instructions, pipetted into a 96 well microplate, and read at 405 nm using the URIT-660 microplate reader (URIT Medical Electronic Co., Ltd, Guangxi, China). Each determination was performed in triplicates.

### Histopathological assessment

The enucleated eyes of the animals were fixed in 10 % phosphate-buffered paraformaldehyde, and embedded in paraffin for histopathological assessment. Sections were made and stained with haematoxylin and eosin and alcian blue [23]. Sections were fixed on glass slides for microscopic examination by a specialist pathologist at the Pathology Department of the Komfo Anokye Teaching Hospital, Kumasi, Ghana.

### Statistical analysis

Results were analysed using one-way analysis of variance followed by Dunnett’s multiple comparisons test using GraphPad Prism (version 5.03; GraphPad, La Jolla, CA). Values were expressed as the mean ± standard error of the mean and *p* ≤ 0.05 was considered statistically significant.

## Results

**Fig. 1:**
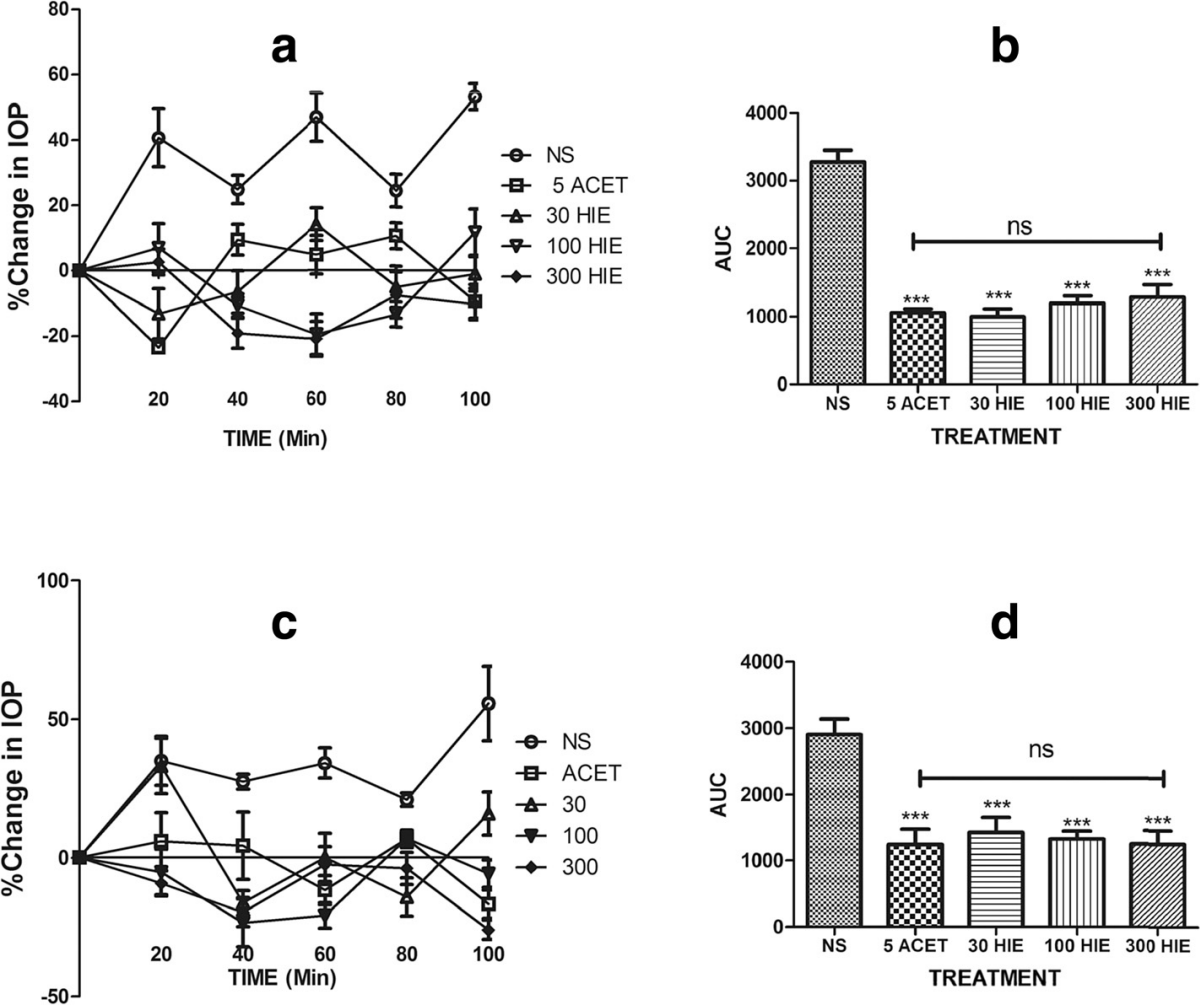
Time-course curves and areas under the curve for the for acute glaucoma study. Time-course curves (**a** & **c**) and areas under the curve (**b** & **d**) for the effects of pretreatment with 30, 100, and 300 mgkg^−1^ of HIE, 5 mgkg^−1^ Acetazolamide (ACET), and 10 mLkg^−1^ normal saline (NS) on Dextrose-induced ocular hypertension of the right eye (**a**, **b**) and left eye (**c**, **d**) in New Zealand White Rabbits. Values plotted represent mean ± SEM (*n* = 5). ***p ≤ 0.001, ANOVA followed by Dunnett’s *post-hoc* test

**Fig. 2:**
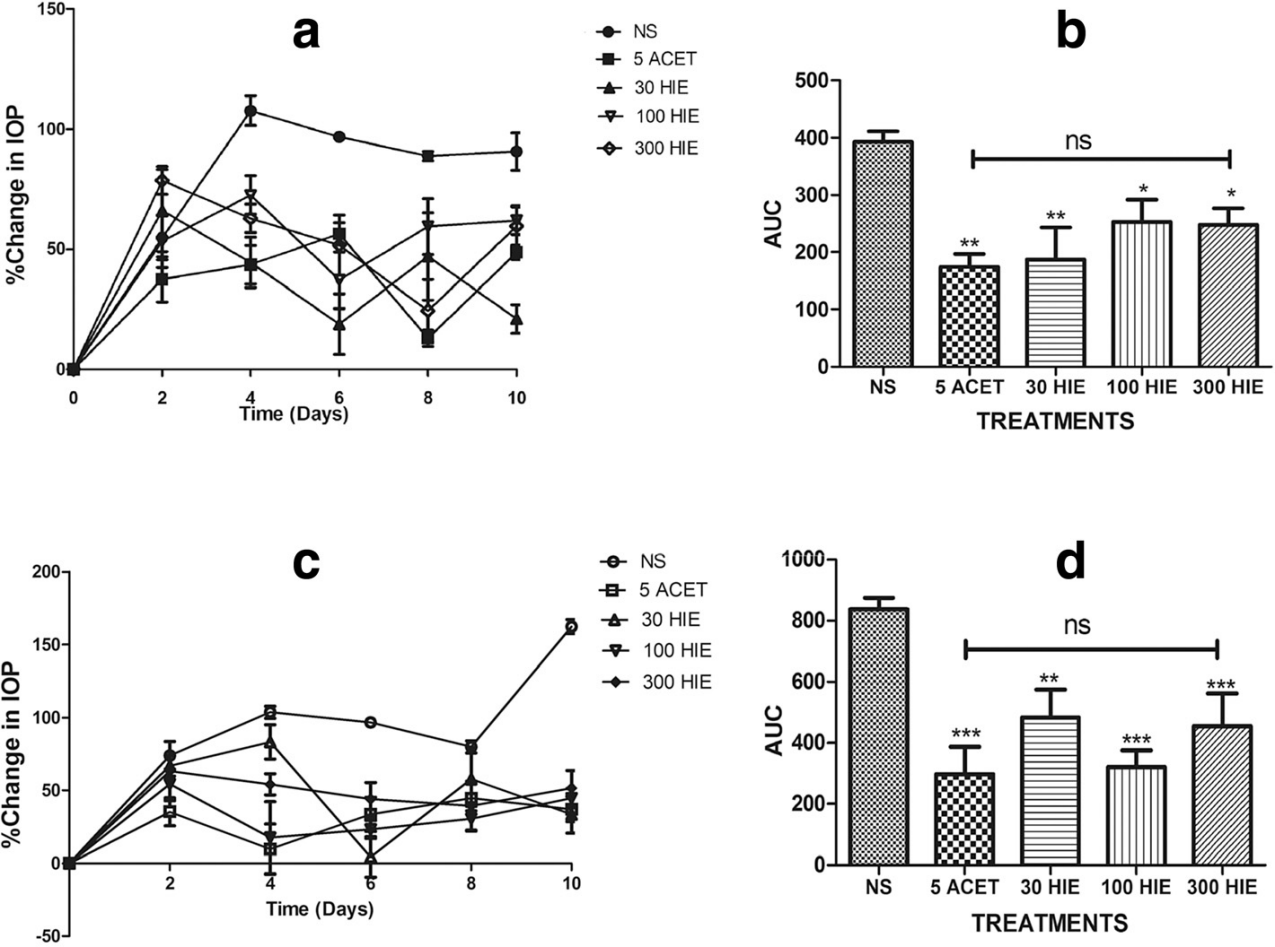
Time-course curves and areas under the curve for the for chronic glaucoma study. Time-course curves (**a** & **c**) and areas under the curve (**b** & **d**) for the effects of treatment with 30, 100, and 300 mgkg^−1^ of HIE, 5 mgkg^−1^ Acetazolamide (ACET), and 10 mLkg^−1^ normal saline (NS) on steroid-induced ocular hypertension of the right eye (**a**, **b**) and left eye (**c**, **d**) in New Zealand White Rabbits. Values plotted represent mean ± SEM (*n* = 5). ***p ≤ 0.001, **p ≤ 0.01, *p ≤ 0.05. ANOVA followed by Dunnett’s *post-hoc* test

HIE pretreatment significantly (*p* ≤ 0.001) prevented the expected rise in IOP in dextrose-induced ocular hypertension compared to the normal saline pre-treated rabbits (Fig. 1); the effect was comparable to acetazolamide pretreatment (*p* ≤ 0.001) as there was no significance (*p* > 0.05) in the IOP lowering effect of acetazolamide and HIE. Similarly, oral treatments of steroid-induced ocular hypertension with HIE showed a significant (*p* ≤ 0.05–0.001) reduction in IOPs of the right and the left eyes of the rabbits vs. normal saline treated animals (Fig. 2). Effects were comparable (*p* ≤ 0.001) to acetazolamide treatment.

### Level of glutathione in aqueous humour

The HIE and acetazolamide treatments in the chronic model of glaucoma studies significantly (*p* ≤ 0.01–0.001) reduced oxidative stress by preserving aqueous endogenous glutathione levels (Table 1).

**Table 1:** Total glutathione (GSH) in the aqueous humour and glutamate levels in the vitreous of controls and HIE-treated chronic ocular hypertensive New Zealand White Rabbits

Treatment	GSH (μM)	Glutamate (nmol)
Normal Saline	3.703 ± 0.437	8.650 ± 0.203
Acetazolamide	11.52 ± 1.171***	7.383 ± 0.130***
30 mgkg^−1^ HIE	10.19 ± 0.632***	6.850 ± 0.092***
100 mgkg^−1^ HIE	7.507 ± 0.760**	7.767 ± 0.010**
300 mgkg^−1^ HIE	11.23 ± 0.316***	6.033 ± 0.257***

Values represent mean ± SEM (*n* = 5). **p ≤ 0.01, ***p ≤ 0.001, one-way analysis of variance followed by Dunnett’s *post hoc* test

### Concentration of glutamate in Vitreous humour

Treatment with HIE and acetazolamide caused a significant (*p* ≤ 0.01–0.001) reduction of excitotoxin in the vitreous humour of the ocular hypertensive treated animals (Table 1).

### Histopathological assessment

**Fig. 3:**
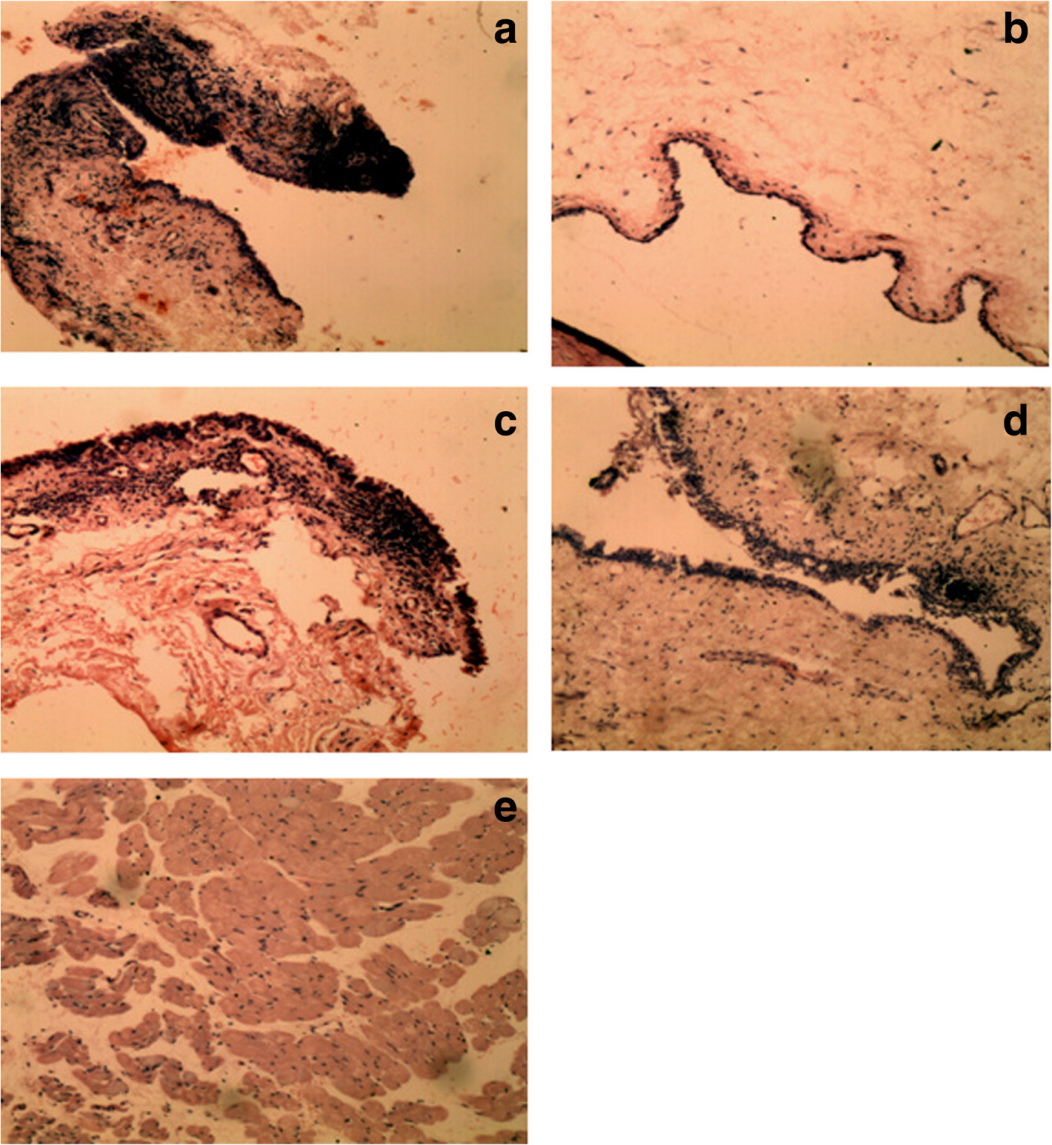
Photomicrographs of the anterior chamber of ocular hypertensive rabbits per the various treatments. Photomicrograph of anterior chamber of rabbits (H and E × 100), (**a**) glaucomatous rabbit with 10 mlkg^−1^ normal saline treatment (Control) showing intense neutrophilic infiltration in the ciliary body, (**b**) glaucomatous rabbit with 5 mgkg^−1^ Acetazolamide treatment. Normal marginal zone of the ciliary process with normal architecture is shown, (**c**) glaucomatous rabbit with 30 mgkg^−1^ HIE treatment indicating moderate neutophilic infiltration in the cilairy body, (**d**) Glaucomatous rabbit with 100 mgkg^−1^ HIE treatment indicating mild neutophilic infiltration and (**e**) glaucomatous rabbit with 300 mgkg^−1^ HIE treatment. There is moderate oedema of the cliary body

The histopatological assessment of the structures of the anterior chamber did indicate relatively reduced signs of morphological changes in ciliary bodies of all rabbits treated with HIE, and acetazolamide. However, there were histopathological signs of tissue alteration characterized by mononuclear infiltration into the ciliary body (Fig. 3).

## Discussion

Glaucoma is described as an assemblage of ocular disorders with multi-factorial causes united by a clinically characteristic optic neuropathy with or without a rise in intraocular pressure (IOP). As it is not a single disease entity, it is sometimes referred to as “the glaucomas” [3] for which IOP reduction remains the only evidence-based treatment approach. Experimental glaucoma is a model that mimics the human condition, and is very useful in studies aimed at understanding the pathophysiology of the disease and in pre-clinical studies of potential anti-glaucoma agents [24].

Acute glaucoma was induced in rabbits using 5 % dextrose administered intravenously; this method has obvious advantages over the water-loading model [25]. Intravenously administered dextrose lowers serum osmolarity after the sugar has been cleared from circulation. This reduced serum osmolarity leads to the movement of water into the eye thereby increasing IOP [24, 25]. Pre-treatment with the extract prevented the expected rise in IOP (*p* ≤ 0.001) compared to the normal saline pretreated group, indicating that the extract could be acting by reducing aqueous humour production or increasing outflow facility [26]. A previous study that assessed the hypotensive effect of *H. indicum* extract on systemic hypertension indicated that it exerts its hypotensive effect via muscarinic receptor stimulation [27], implying that its ocular hypotensive effect could be due to enhanced outflow facility rather than reduced aqueous humour production. Again, an important relation has been found between systemic blood pressure and the development of glaucoma. That is, an increased blood pressure as in the case of hypertension impairs autoregulation of blood flow, which consequentially affects blood circulation to the optic nerve inducing glaucoma via ischemic tendencies [28]. On the other hand, hypotension has also been named as a risk factor for glaucoma therefore, further studies would be needed to ascertain the clinical application of HIE in glaucoma management as it has been reported to reduce blood pressure in some studies 48.25 ± 3.56 % [27, 29].

The anti-glaucoma potential of HIE was further substantiated by testing its ocular hypotensive effect on a more sustained (chronic) model of ocular hypertension. The corticosteroid–induced model bears semblance of POAG, and is characterized by aqueous outflow obstruction, optic nerve cupping and visual field defects [22, 30]. HIE treatment reduced (*p* ≤ 0.05–0.001) the IOP induced by steroid pretreatment in the rabbits. A recent study showed that POAG, as modelled by corticosteroid-induced ocular hypertension in rabbits, is a multi-tissue disease entity involving the trabecular meshwork, the optic nerve head, the lateral geniculate nuclei, and the visual cortex. Stressors such as repeated steroid intake triggers oxidative stress resulting in compromised aqueous humour antioxidant system and apoptotic trabecular meshwork cell loss. This apoptotic cell loss is informed by severe mitochondrial damage altering tissue function and integrity [31]. The extract could therefore be exerting its ocular hypotensive effect via improving aqueous outflow, protection of the structural integrity of the trabecular meshwork or both [31]. The reference drug, acetazolamide, on the other hand, is a specific carbonic anhydrase inhibitor that lowers the intraocular pressure of mammal’s eyes by partially inhibiting aqueous humour formation [32]. In addition to the inhibition of aqueous production, it has been reported to also decrease oxidative damage of the trabecular meshwork and more so in the presence of active mitochondria [33]. Mitochondria are predicted as the key intracellular target for most drugs with antioxidant properties [34]. This is suggestive of the possibility of multiple mechanistic pathways in exerting their therapeutic effect. Acetazolamide is one of the few medications that exist in both oral and topical forms that are effective in reducing IOP and improving retinal blood flow [35]. Its oral formulation affords ophthalmic caregivers the options of achieving greater bioavailability (oral bioavailability of more than 90 %) for aggressive forms of glaucoma when the short precorneal residence time poses the challenge of poor bioavailability upon topical application [36]. However, oral doses of acetazolamide are associated with a myriad of systemic side effects due to the wide distribution of the carbonic anhydrase enzyme, which has many functions including transporting CO_2_ from the tissues to the lung, excreting and reabsorbing electrolytes and H^+^ ions in the kidney, secreting H^+^ ions into the gastric mucosa, and maintaining the major buffer system of the human body [37, 38]. Preliminary data from our laboratory indicates that topical application of HIE into the conjunctival cul-de-sac is safe, but medium term oral (subchronic) usage of therapeutic doses produced subtle morphometric changes in the liver, kidney and the spleen upon histological assessment. Further studies are still ongoing in this regard.

It is clear that oxidative stress, which is an important etiologic factor in the pathogenesis of glaucoma, [39] is manly driven by free radicals in living systems when endogenous antioxidant defences are deficient [40]. It is proven in humans that both elevated IOP and visual field loss are notably related to the amount of oxidative DNA damage affecting trabecular meshwork (TM) cells, thereby affecting outflow facility [41]. Glutathione has been found in significant proportions in the aqueous humour and plays an essential role in defending the system against oxidative stress-provoked diseases [42]. The antioxidant statuses of biological samples are therefore useful as a marker of oxidative stress [43]. The extract treatment preserved endogenous aqueous humour glutathione levels (*p* ≤ 0.01–0.001), which suggests its usefulness not only in reducing IOP, but also in providing a protection against oxidative damage critical in advancing the progression of glaucomatous neurodegeneration. This presupposes that the HIE targets mitochondrial cells of the trabecular meshwork in exerting its effect.

Excitotoxicity elicited by the amino acid glutamate is gaining attention so far as the mediation of neuronal death in many disorders. An understanding of excitotoxic injury provides clues in the search for answers to such fundamental questions such as the continual loss of retinal ganglion cells despite achieving IOP control [44]. Amidst the ranging controversy over its pathogenic role in glaucoma [45, 46], there was an observed reduction of glutamate concentration (*p* ≤ 0.01–0.001) in the extract-treated rabbits. Studies have shown that vitreous is easily obtainable and remains an important biological sample in postmortem analysis, in that it is less prone to putrefaction and contamination relative to other body fluids as postmortem biochemical changes occur more slowly in the eye [47]. The hypothesized association between glutamate excitotoxicity and neurological disorders such as glaucoma was well managed by the HIE treatment [48, 49].

Histopathological changes were remarkable in the anterior chamber of normal saline treated animals but relatively minimal in the extract-treated and the acetazolamide-treated rabbits. Glaucoma is exceptional amongst ocular disorder in that its principal pathophysiology involves structures in both the anterior and posterior segments of the eye. This affords the option of tracking pathological changes in either segment or both [23].

The extract owes its net anti-glaucoma potential effect to the synergistic effect of its phytochemicals acting concomitantly on the diverse etiologic factors. Other researchers have established that some alkaloids possess hypotensive effect more particularly via muscarinic action [50]. Saponins have also been demonstrated to have some hypotensive activity [51]. Flavonoids, in general, have been proven to possess antioxidant activity relevant for free scavenging activity that is necessary to preserve the eye’s endogenous antioxidant system [52]. This cocktail of bioactive compounds detected in HIE affirms the mechanistic multiplicity of its therapeutic effect in experimental glaucoma management.

## Conclusion

The aqueous whole plant extract of *H. indicum* exhibits ocular hypotensive, antioxidant and potential neuroprotective effects hence, it could be a useful anti-glaucoma drug with further studies.

### Availability of supporting data

Supporting data are all available in this study.
